# Metagenome sequencing and 98 microbial genomes from Juan de Fuca Ridge flank subsurface fluids

**DOI:** 10.1038/sdata.2017.37

**Published:** 2017-03-28

**Authors:** Sean P. Jungbluth, Jan P. Amend, Michael S. Rappé

**Affiliations:** 1 Center for Dark Energy Biosphere Investigations, University of Southern California, Los Angeles, California 90089, USA; 2 Department of Earth Sciences, University of Southern California, Los Angeles, California 90089, USA; 3 Department of Biological Sciences, University of Southern California, Los Angeles, California 90089, USA; 4 Hawaii Institute of Marine Biology, SOEST, University of Hawaii, Kaneohe, Hawaii 96744, USA

**Keywords:** Metagenomics, Marine biology, Genome informatics, Microbial ecology, DNA sequencing

## Abstract

The global deep subsurface biosphere is one of the largest reservoirs for microbial life on our planet. This study takes advantage of new sampling technologies and couples them with improvements to DNA sequencing and associated informatics tools to reconstruct the genomes of uncultivated Bacteria and Archaea from fluids collected deep within the Juan de Fuca Ridge subseafloor. Here, we generated two metagenomes from borehole observatories located 311 meters apart and, using binning tools, retrieved 98 genomes from metagenomes (GFMs). Of the GFMs, 31 were estimated to be >90% complete, while an additional 17 were >70% complete. Phylogenomic analysis revealed 53 bacterial and 45 archaeal GFMs, of which nearly all were distantly related to known cultivated isolates. In the GFMs, abundant Bacteria included Chloroflexi, Nitrospirae, Acetothermia (OP1), EM3, Aminicenantes (OP8), Gammaproteobacteria, and Deltaproteobacteria, while abundant Archaea included Archaeoglobi, Bathyarchaeota (MCG), and Marine Benthic Group E (MBG-E). These data are the first GFMs reconstructed from the deep basaltic subseafloor biosphere, and provide a dataset available for further interrogation.

## Background & Summary

Beneath the sediments of the deep ocean, the subseafloor igneous basement presents a largely unexplored habitat that likely plays a crucial role in global biogeochemical cycling^1^. This system also provides a gradient of untapped environments for the discovery of novel microbial life. Because of extensive hydrothermal circulation, the porous uppermost igneous crust is likely quite suitable for microbial life^2^. Entrainment of deep seawater into young ridge flanks injects a variety of terminal electron acceptors into the deep ocean crust, establishing chemical gradients with the reducing deeper fluids, and thereby fueling redox-active elemental cycles^3^. The redox disequilibria and circulation of fluids through the permeable network of volcanic rock sustains a largely uncharacterized microbial community that potentially extends thousands of meters below the seafloor^4^. In such environments, temperatures may be elevated and energy and nutrients may be limited, providing a unique combination of challenges to microbial life.

**Figure 1:**
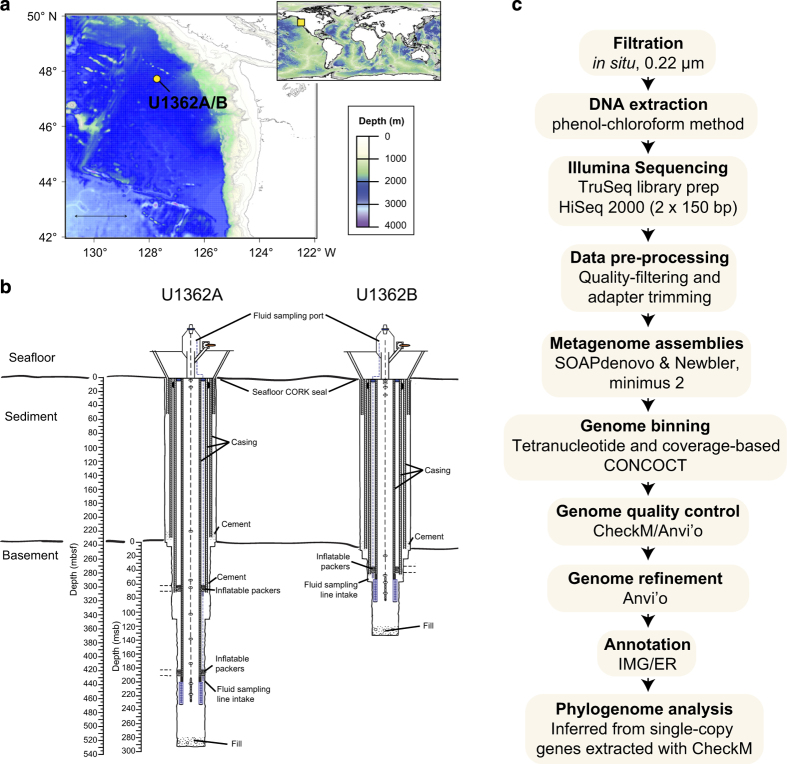
Sampling and methods used for this study. (**a**) Bathymetric map of Juan de Fuca Ridge boreholes U1362A and U1362B with inset world map showing region location. (**b**) Schematic of CORK observatories at U1362A and U1362B. (**c**) Workflow used to process basement crustal fluid samples to generate metagenomes and GFMs.

CORK (circulation obviation retrofit kit) observatories have been used to collect warm, anoxic crustal fluids originating from boreholes drilled into 1.2 and 3.5 million-year-old ridge flank of the Juan de Fuca Ridge (JdFR)^5^. This young, hydrologically-active basaltic crustal environment is overlain by a thick (>100 m) blanket of sediment that serves to locally restrict fluid circulation in the ocean basement^6,7^. The sampling and interrogation of raw basement fluids enabled by CORK observatories has revealed the presence of novel microbial lineages that are related to uncultivated candidate microbial phyla with unknown metabolic characteristics^8–11^. Here, we present the genomes from metagenomes (GFMs) of two pristine large-volume igneous basement fluid samples collected from JdFR flank CORK observatories within boreholes U1362A and U1362B (Fig. 1).

Shotgun sequencing produced 503 and 705 megabase pairs (Mbp) of unassembled sequence data from individual borehole U1362A and U1362B samples (Table 1). The metagenomes were assembled separately into 137,575 and 212,307 scaffolds totaling 170 and 168 Mbp of sequence data from U1362A and U1362B, respectively (Tables 1 and 2). The maximum scaffold lengths constructed from U1362A and U1362B metagenome were, 541 and 1,137 Mbp, respectively (Table 2). The success of this assembly to generate long scaffolds that represent large, intact fractions of individual genomes provides a significant foundation for which to apply binning methods to piece together genomes from populations in the original samples.

Several methods were used to generate GFMs, which were then evaluated, further curated, and reduced to a set for additional characterization. Ultimately, analysis was performed on 98 GFMs that were over 200 Kbp in length, contained marker gene sets identified by CheckM, and were >10% complete (Table 3 and Supplementary Table 1).

**Figure 2:**
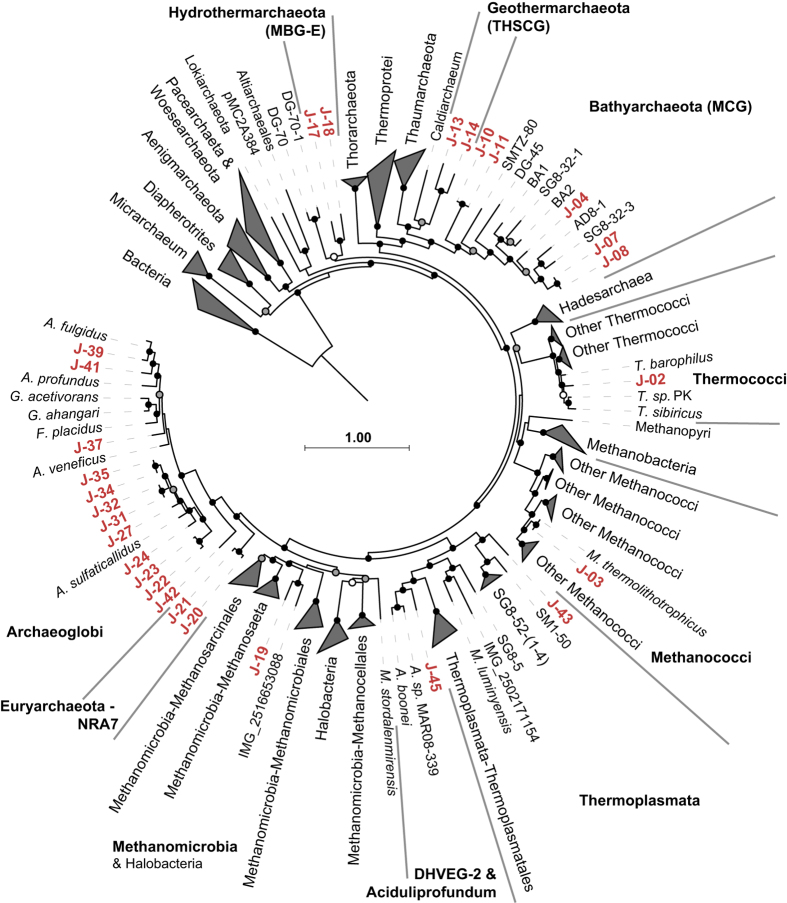
Phylogenomic relationships between archaeal genomes >50% complete identified in CORK borehole fluid metagenomes and other closely related genomes. The scale bar corresponds to 1.00 substitutions per amino acid position. Some groups are collapsed to enhance clarity and all groups with taxonomic identities are shown. The names of major lineages with GFMs found in Juan de Fuca Ridge basement fluids are indicated with bold-face font. JdFR GFM prefixes are abbreviated from ‘JdFR’ to ‘J’ and labeled using red-colored text. Black (100%), gray (≥80%), and white (≥50%) circles indicate nodes with high local support values, from 1,000 replicates.

**Figure 3:**
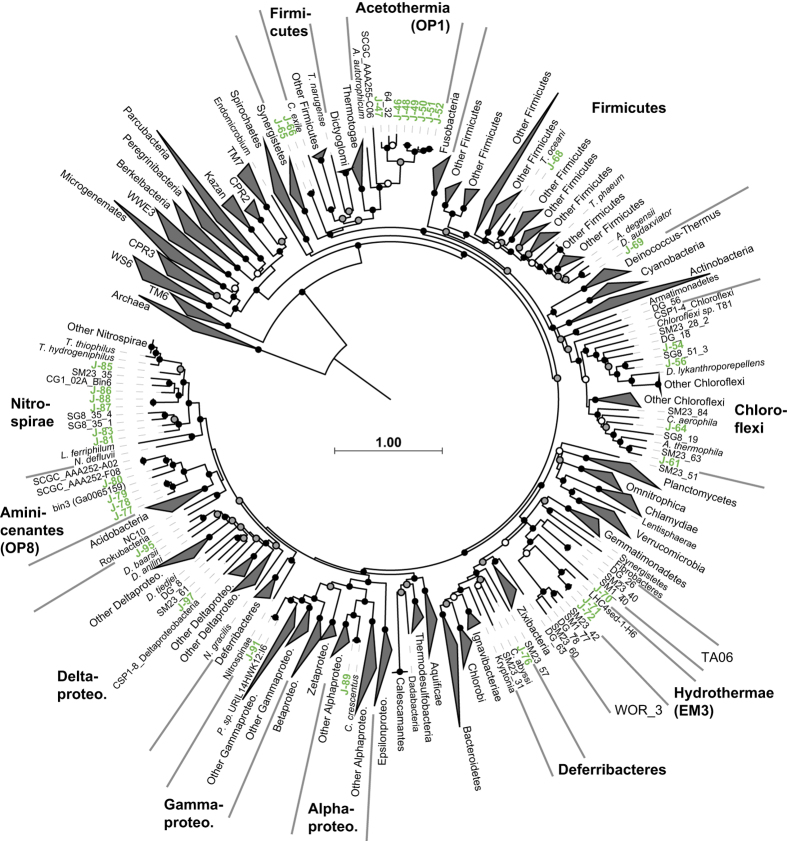
Phylogenomic relationships between bacterial genomes >50% complete identified in CORK borehole fluid metagenomes and other closely related genomes retrieved from popular databases. JdFR GFM prefixes are labeled using green-colored font. Other information as in Fig. 2.

Phylogenetic analysis of concatenated universally conserved marker gene alignments (Figs 2 and 3,Supplementary Figs 1 and 2) and taxonomic identification of SSU rRNA genes (Table 4 (available online only)) allowed for the phylum-level identification of most of the 53 bacterial and 45 archaeal GFMs. The U1362A and U1362B borehole fluid GFMs were comprised of many of the same microbial lineages described previously using SSU rRNA sequencing^8,11^, including bacterial groups Chloroflexi (11), Nitrospirae (8), Acetothermia (OP1; 7), EM3 (5), Aminicenantes (OP8; 4), Gammaproteobacteria (4), and Deltaproteobacteria (4), and archaeal groups Archaeoglobi (21), Bathyarchaeota (MCG; 9), and Marine Benthic Group E (MBG-E; 3) (Table 5 (available online only) and Supplementary Table 1). In this study, we identified the first near-complete genomes from archaeal and bacterial lineages THSCG, MBG-E, and EM3 and, based on the warm, subsurface and hydrothermally-associated environments from which these groups tend to be found, propose the names Geothermarchaeota, Hydrothermarchaeota, and Hydrothermae, respectively.

The 98 genomes described here were deposited into the National Center for Biotechnology Information (NCBI) and Integrated Microbial Genomes (IMG) databases^12^. The genome data described here are the first GFMs described from the deep subseafloor volcanic basement environment and will be used to interrogate the functional underpinnings of individual microbial lineages within this remote and distinct ecosystem. Considering that genome binning methods cannot yield comprehensive segregation of all entities in complex samples^13^, and that informatics tools are continuously improving, we recommend that anyone using these data verify the contents of these GFMs with the latest tools available.

## Methods

### Borehole fluid sampling

Sample collection methods are described elsewhere^11^. Briefly, during R/V Atlantis cruise ATL18-07 (28 June 2011-14 July 2011) samples of basement crustal fluids were collected from CORK observatories located in 3.5 million-year-old ocean crust east of the Juan de Fuca spreading center. Basement fluids were collected from lateral CORKs (L-CORKs) at boreholes U1362A (47°45.6628′N, 127°45.6720′W) and U1362B (47°45.4997′N, 127°45.7312′W) via polytetrafluoroethylene (PTFE)-lined fluid delivery lines that extend to 200 (U1362A) and 30 (U1362B) meters sub-basement. Fluids were filtered in situ through Steripak-GP20 (Millipore, Billerica, MA, USA) polyethersulfone filter cartridges containing 0.22 μm pore-sized membranes using a mobile pumping system. Filtration rates were 1 l/min in laboratory trials, indicating that ~124 liters and ~70 liters were filtered from boreholes U1362A and U1362B, respectively.

### Metagenomic DNA sequencing

Borehole fluid nucleic acids were extracted using a modified phenol/chloroform lysis and purification method and is described in detail elsewhere^11^. The samples used in this study correspond to samples SSF21–22 (U1362A) and SSF23–24 (U1362B) labelled by Jungbluth *et al.*^11^. Library preparation and sequencing was conducted by the Department of Energy Joint Genome Institute as part of the Community Science Program. A total of 100 ng (U1362A) or 5 ng (U1362B) of DNA was sheared using a focused-ultrasonicator (Covaris, Woburn, MA, USA). The sheared DNA fragments were size selected using SPRI beads (Beckman Coulter, Brea, CA, USA). The selected fragments from U1362A were then end-repaired, A-tailed, and ligated of Illumina compatible adapters (Integrated DNA Technologies, Coralville, IA, USA) using KAPA-Illumina library creation kit (KAPA Biosystems, Wilmington, MA, USA). The selected fragments from U1362B were treated with end repair, ligation of adapters and 9 cycle of PCR on the Mondrian SP+ Workstations (Nugen, San Carlos, CA, USA) using the Ovation SP+ Ultralow DR Multiplex System kit (Nugen).

The library was quantified using KAPA Biosystem’s next-generation sequencing library qPCR kit and run on a LightCycler 480 real-time PCR instrument (Roche, Basel, Switzerland). The quantified U1362A library was then prepared for sequencing on the HiSeq sequencing platform (Illumina, San Diego, CA, USA) utilizing a TruSeq paired-end cluster kit, v3, and Illumina’s cBot instrument to generate clustered flowcell for sequencing. The U1362B library was prepared for sequencing in the same manner except the library was multiplexed with one other sample library prior to use of the TruSeq kit. Sequencing of the flowcell was performed on the Illumina HiSeq2000 sequencer using a TruSeq SBS sequencing kit 200 cycles, v3, following a 2×150 indexed run recipe.

Insert size analysis was performed at JGI using bbmerge to pair overlapping reads and, with sufficient coverage, non-overlapping reads using gapped kmers. The ‘percentage reads joined’ was calculated by (number of joined reads/total number of reads×100). Raw reads were used for the insert size calculation (no trimming or filtering). Insert size statistics for the U1362A metagenome were: 68.342% reads joined, 216.60 bp average read length, 37.40 bp s.d. read length, and 215 bp mode read length. Insert size statistics for the U1362B metagenome were: 50.40% reads joined, 210.80 bp average read length, 39.70 bp s.d. read length, and 196 bp mode read length.

### Metagenome quality control, read trimming and assembly

Assembly was performed by the JGI; corresponding JGI assembly identifications are 1,020,465 (U1362A) and 1,020,462 (U1362B). Raw Illumina metagenomic reads were screened against Illumina artifacts with a sliding window with a kmer size of 28, step size of 1. Screened read portions were trimmed from both ends using a minimum quality cutoff of 3, reads with 3 or more ‘Ns’ or with average quality score of less than Q20 were removed. In addition, reads with a minimum sequence length of <50 bp were removed. Trimmed, screened, paired-end Illumina reads were assembled using SOAPdenovo version 1.05 (ref. 14) with default settings (options: -K 81, -p 32, -R, -d 1) and a range of Kmers (81, 85, 89, 93, 97, 101). Contigs were generated by each assembly were de-replicated and sorted into two pools based on length. Contigs smaller than 1,800 bp were assembled using Newbler version 2.7 (Life Technologies, Carlsbad, CA, USA) in an attempt to generate larger contigs (flags: -tr, -rip, -mi 98, -ml 80). All assembled contigs larger than 1,800 bp were combined with the contigs generated from the final Newbler run using minimus2 (AMOS) version 3.1.0 (ref. 15) (flags: -D MINID=98 -D OVERLAP=80). JGI-reported read depths available in IMG were estimated based on read mapping with JGI custom mapping programs.

### Gene prediction and annotation

All aspects of metagenome annotation performed at JGI have been described previously^12^ and can be found at https://img.jgi.doe.gov/m/doc/MGAandDI_SOP.pdf. Briefly, metagenome sequences were preprocessed to resolve ambiguities, trim low-quality regions and trailing ‘N’s using LUCY^16^, masked for low-complexity regions using DUST^17^, and dereplicated (95% threshold). Genes were predicted in the following order: CRISPRs, non-coding RNA genes, protein-coding genes. CRISPR elements were identified by concatenating the results from the programs CRT^18^ and PILER-CR^19^. tRNAs were predicted using tRNA scan SE-1.23 (ref. 20) three times using each of the domains of life (Bacteria, Archaea, Eukaryota) as the parameter required; the best scoring predictions were selected. Fragmented tRNAs were identified by comparison to a database of tRNAs identified in isolate genomes. Ribosomal RNA genes were predicted using JGI-developed rRNA models (SPARTAN: SPecific & Accurate rRNA and tRNA ANnotation). Protein-coding genes were identified using a majority rule-based decision schema using four different gene callings tools: prokaryotic GeneMark (hmm version 2.8)^21^, MetaGene Annotator version 1.0 (ref. 22), Prodigal version 2.5 (ref. 23), and FragGeneScan version 1.16 (ref. 24). When there was no clear decision, the selection was based on preference order of gene callers determined by JGI-based runs on simulated metagenomic datasets [GeneMark > Prodigal > Metagenome > FragGeneScan].

Predicted CDSs were translated and associated with Pfams, COGs, KO terms, EC numbers, and phylogeny. Genes were associated with Pfam-A using hmmsearch^25^. Genes were associated with COGs by comparing protein sequences with the database of PSSMs for COGs downloaded from NCBI; rpsblast v2.26 (ref. 26) was used to find hits. Assignments of KO terms, EC numbers, and phylogeny were made using similarity searches to reference databases constructed by starting with the set of all non-redundant sequences taken from public genomes in IMG. Sequences from the KEGG database that were not present in IMG were added and all data was merged to related gene IDs to taxa, KO terms, and EC numbers. USEARCH v6.0.294 (ref. 27) was used to compare predicted protein-coding genes to genes in this database and the top five hits for each gene were retained. Phylogenetic assignment was based on the top hit only; for assignment of KO terms, the top five hits to genes in the KO index were used. A hit resulted in an assignment if there was at least 30% identity and greater than 70% of the query protein sequence or the KO gene sequence were covered by the alignment.

### Genome binning

Assemblies from the U1362A and U1362B metagenomes were combined and used to generate GFMs. Four different genome binning approaches were used to identify the workflow that yielded the most favorable balance between maximizing genome completeness while minimizing contamination for these metagenomes: MaxBin^28^, ESOM^29^, MetaBAT^30^, and CONCOCT^31^.

Genome binning was performed using MaxBin version 2.1.1 (ref. 28) with the 40 marker gene set universal among Bacteria and Archaea^32^, minimum scaffold length of 2,000 bp, and default parameters. Scaffold coverage from each metagenome was estimated using the quality-control filtered raw reads as input for mapping using Bowtie2 version 2.2.3 (ref. 33) used within MaxBin.

Genome binning was also performed using a combination of tetranucleotide frequencies and differential coverage in emergent self-organizing maps (ESOM)^29^. Scaffold coverage was calculated using bbmap version 35.40 and the jgi_summarize_bam_contig_depths script from the MetaBAT pipeline^30^. Scripts downloaded from (http://github.com/tetramerFreqs/Binning) were used to calculate tetramer frequencies and create input files for ESOM. A robust Z-transformation was applied to the input data prior to generation of the ESOM. Scaffolds 10 Kbp or greater were cut into fragments of 2,000 bp prior to clustering. The number of epochs used for clustering was 20 and the dimensions of the ESOM were 400×430 (Supplementary Fig. 3).

Using MetaBAT version 0.26.3 (ref. 30), genome binning was performed with the jgi_summarize_bam_contig_depths script and the same scaffold coverage map calculated using bbmap described above. Default parameters were used.

Finally, genome binning was performed using CONCOCT^31^ within the Anvi’o package, version 1.1.0 (ref. 34). The metagenomic workflow employed here is described online (merenlab.org/2015/05/02/anvio-tutorial), and included as input data the quality-filtered raw sequence reads from both metagenomes, as well as assemblies generated by the JGI. The scaffold coverage map was calculated using bbmap version 35.82. Scaffolds greater or equal to 2.5 Kbp were used for binning with CONCOCT.

### Comparison of genome binning methods and bin curation

Completeness and contamination of all GFMs created using the four binning methods were assessed using CheckM version 1.0.5 (ref. 35). Compared to the GFMs generated via MaxBin, ESOM, and MetaBAT, GFMs generated with CONCOCT had the highest average percent completeness for bins that were at least 50% complete (Table 3). Genome completeness was the primary criterion used in the selection of the binning method because the facilitated supervised binning via the ‘anvi-refine’ function in Anvi’o proved an effective means to remove contamination from a draft set of genome scaffolds. Manual refinements to the GFMs were executed in Anvi’o using differential coverage, tetranucleotide frequency, and marker gene content (i.e., completeness/contamination). Bin splitting was assisted by the analysis of SSU rRNA genes identified using CheckM and inspected via the SILVA/SINA online aligner version 1.2.11 (ref. 36) with the following parameters: minimum identity with query sequence, 0.8, and number of neighbors per query sequence, 3. When SSU rRNA genes of different taxonomic origin were found to conflict within a single bin, those bins were further scrutinized and split manually. In most instances where contamination was >50%, splitting bins into their U1362A and U1362B components resolved conflicts. Bins were split until no SSU rRNA gene conflicts remained and all bins had been manually inspected and screened for outlying scaffolds. Four other marker gene sets^31,37–39^ were used to compare completeness and contamination within Anvi’o (Supplementary Fig. 4). A total of 252 GFMs were identified after curation with Anvi’o, and completeness and contamination of the final GFMs was ultimately estimated with CheckM and the marker gene set of Wu and colleagues^32^. Of these, 98 were at least 10% complete (Table 5 (available online only) and Supplementary Table 1), which was used as a minimum cutoff because the GFMs all contained marker genes that allowed them to be assigned phylogenetic identities via CheckM. The 98 GFMs included a total of 16,066 scaffolds and 154,609,643 bp.

### Phylogenomics and identification of genomes from metagenomes

From all genomes described here with completeness >10% and relevant GFMs and single-amplified genomes (SAGs) from the Integrated Microbial Genomes (IMG)^40^, ggKbase, and National Center for Biotechnology Information (NCBI) GenBank databases, phylogenetically informative marker genes were identified and extracted using the ‘tree’ command in CheckM. In CheckM, open reading frames were called using prodigal version 2.6.1 (ref. 23) and a set of 43 lineage-specific marker genes, similar to the universal set used by PhyloSift^41^, were identified and aligned using HMMER version 3.1b1 (ref. 42). The 61 GFMs with >50% completeness were assigned taxonomic identifications through analysis of a concatenated marker gene alignment (6,988 amino acid positions) and placement in a phylogenomic tree with related GFMs and SAGs found in the NCBI, IMG, and ggKbase databases. The phylogeny was produced using FastTree version 2.1.9 (ref. 43) with the WAG amino acid substitution model and ‘fastest’ mode. Bootstrap values reported by FastTree analysis indicate local support values. To leverage the taxonomic identifications assigned to GFMs with >50% completeness to assist in the identification of 37 GFMs with completeness 10–50%, an additional phylogenetic analysis with only the 98 Juan de Fuca GFMs was performed in ARB^44^ using RAxML version 7.7.2 (ref. 45) with the PROTGAMMA rate distribution model and WAG amino acid substitution model. Bootstrapping was executed in ARB using the RAxML rapid bootstrap analysis algorithm^46^ with 100 bootstraps. To further aid in identification of GFMs, SSU rRNA genes were extracted from 49 genome bins using the ‘ssu_finder’ command within CheckM and identified via the SILVA/SINA online aligner version 1.2.11 (ref. 36) with the version 123 database and the following parameters: minimum identity with query sequence, 0.8, and number of neighbors per query sequence, 3 (Table 4 (available online only)).

## Data Records

The raw Illumina sequencing reads, assembled and annotated metagenomes (Table 1), and 98 GFMs generated from the Juan de Fuca Ridge basement fluids (Table 5 (available online only) and Supplementary Table 1) are available from the NCBI databases (Data Citation 1). FASTA files containing the contigs of all 98 GFMs are available on figshare (Data Citation 2). Text files needed to isolate scaffold sets for all 98 GFMs in IMG/M are available on figshare (Data Citation 3). A FASTA file containing 54 SSU rRNA genes with length >300 base pairs extracted from the 98 GFMs is available on figshare (Data Citation 4). A text file containing all IMG/M annotations associated with the 98 GFMs is available on figshare (Data Citation 5).

## Technical Validation

To assess the completeness and contamination of the genomes, we analyzed the abundance of single copy marker genes present in all bacterial and archaeal GFMs using CheckM^35^ (see Methods for details).

## Usage Notes

The U1362A and U1362B metagenome projects and raw sequencing reads are available via the IMG-M web portal under Taxon ID numbers 330002481 (U1362A) and 3300002532 (U1362B). Gold Analysis Project ID numbers are Ga0004278 (U1362A) and Ga0004277 (U1362B). Sample metadata can be accessed at BioProject (Data Citation 1). The NCBI BioSamples used here are SAMN03166137 (U1362A) and SAMN03166138 (U1362B). FASTA files containing the contigs of all 98 genomes from metagenomes can be accessed at Data Citation 2. IMG/M-relevant files needed to isolate scaffold sets for all 98 genomes from metagenomes can be accessed at Data Citation 3. A FASTA file containing 54 SSU rRNA genes with length >300 base pairs extracted from the 98 genomes from metagenomes can be accessed in Data Citation 4. IMG/M annotations associated with the scaffolds of all 98 genomes from metagenomes can be accessed at Data Citation 5. The GFMs can be accessed via the National Center for Biotechnology Information (NCBI) using the BioSample and GenBank accessions provided in Table 5 (available online only) and Supplementary Table 1.

## Additional Information

**How to cite this article:** Jungbluth, S. P. *et al.* Metagenome sequencing and 98 microbial genomes from Juan de Fuca Ridge flank subsurface fluids. *Sci. Data* 4:170037 doi: 10.1038/sdata.2017.37 (2017).

**Publisher’s note:** Springer Nature remains neutral with regard to jurisdictional claims in published maps and institutional affiliations.

## Supplementary Material



Supplementary Figures

Supplementary Table 1

## Figures and Tables

**Table 1:** Metagenome sequencing statistics reported in IMG.

	**U1362A**			**U1362B**		
	**No. assembled (% of assembled)**	**No. unassembled (% of unassembled)**	**Total (% of total)**	**No. assembled (% of assembled)**	**No. unassembled (% of unassembled)**	**Total (% of total)**
Number of sequences	137,575 (8.08)	1,564,185 (91.92)	1,701,760 (100)	212,307 (7.60)	2,582,305 (92.40)	2,794,612 (100)
Number of bases	169,908,118 (33.78)	333,077,167 (66.22)	502,985,285 (100)	168,044,831 (23.83)	537,213,224 (76.17)	705,258,055 (100)
GC count	82,941,377 (48.82)	163,998,454 (49.24)	246,939,831 (49.09)	87,552,944 (52.10)	270,739,112 (50.40)	35,829,2056 (50.80)
	Genes					
rRNA genes	609 (0.22)	1,124 (0.08)	1,733 (0.10)	682 (0.21)	1,219 (0.05)	1,901 (0.07)
*16S rRNA*	198 (0.07)	162 (0.01)	360 (0.02)	199 (0.06)	191 (0.01)	390 (0.01)
*23S rRNA*	315 (0.12)	617 (0.04)	932 (0.05)	359 (0.11)	587 (0.02)	946 (0.04)
Protein coding genes	267,511 (98.50)	1,489,984 (99.63)	1,757,495 (99.46)	319,764 (98.87)	2,344,253 (99.37)	2,664,017 (99.31)
*with Product Name*	160,006 (58.91)	438,495 (29.32)	598,501 (33.87)	170,964 (52.86)	559,698 (23.73)	730,662 (27.24)
with COG	186,319 (68.60)	675,287 (45.16)	861,606 (48.76)	207,169 (64.06)	834,581 (35.38)	1,041,750 (38.84)
with Pfam	172,149 (63.38)	519,243 (34.72)	691,392 (39.13)	187,717 (58.04)	647,505 (27.45)	835,222 (31.14)
with KO	131,624 (48.46)	604,486 (40.42)	736,110 (41.66)	151,186 (46.75)	773,722 (32.80)	924,908 (34.48)
with Enzyme (EC)	73,927 (27.22)	356,052 (23.81)	429,979 (24.33)	83,086 (25.69)	440,214 (18.66)	523,300 (19.51)
with MetaCyc	52,288 (19.25)	244,997 (16.38)	297,285 (16.82)	58,809 (18.18)	301,799 (12.79)	360,608 (13.44)
with KEGG	78,361 (28.85)	365,246 (24.42)	443,607 (25.10)	88,171 (27.26)	455,581 (19.31)	543,752 (20.27)

**Table 2:** Metagenome scaffold length statistics.

	**U1362A**		**U1362B**	
**Minimum scaffold length**	Num. of Scaffolds*	Total Scaffold Length*	Num. of Scaffolds*	Total Scaffold Length*
All	137,575	169,908,118	212,307	168,044,831
1 kb	25,958	122,371,000	22,179	94,767,619
2.5 kb	10,118	98,145,686	7,817	72,903,412
5 kb	4,544	78,915,922	3,232	57,281,039
10 kb	1,933	60,882,353	1,339	44,376,823
25 kb	615	41,195,243	435	30,631,998
50 kb	273	29,394,283	191	22,129,275
100 kb	105	18,147,775	72	13,983,109
250 kb	15	5,160,259	11	5,597,623
500 kb	1	540,961	3	2,801,775
1 mb	0	0	1	1,136,825

*Numbers listed are the cumulative sum of all scaffolds equal to or above the scaffold length.

**Table 3:** Genome binning method summary.

**Method**	**Num Bins**	**Num Bins >10% Complete**	**Num Bins >50% Complete**	Avg. Completeness (%)*	Avg. Contamination (%)*
CONCOCT	66	56	46	90.9	50.8
ESOM	60	54	49	90.4	71.5
MaxBin	75	66	51	85.7	42.9
MetaBAT	69	64	45	87.7	9.7
CONCOCT (post manual curation in Anvi’o)	252	98	61	84.4	3.3

*Average calculated for bins >50% completeness.

**Table 4:** Summary of SSU rRNA genes in genome bins

**Genome Bin**	**JGI Scaffold ID**	**Sequence Length (bp)**	**Percent Similarity (%)**	**SILVA Taxonomy (version 123)**
JdFR-03	JGI24020J35080_1000540	910	99.3	Archaea;Euryarchaeota;Methanococci;Methanococcales;Methanococcaceae;Methanothermococcus;
JdFR-04	JGI24020J35080_1005350	527	90.2	Archaea;Miscellaneous Crenarchaeotic Group;
JdFR-06	JGI24019J35510_1000399	793	93.3	Archaea;Miscellaneous Crenarchaeotic Group;
JdFR-07	JGI24019J35510_1000402	874	96.1	Archaea;Miscellaneous Crenarchaeotic Group;
JdFR-08	JGI24020J35080_1000153	627	96.3	Archaea;Miscellaneous Crenarchaeotic Group;
JdFR-10	JGI24019J35510_1000009	1,498	87.0	Archaea;Miscellaneous Crenarchaeotic Group;
JdFR-10	JGI24020J35080_1001231	1,498	87.0	Archaea;Miscellaneous Crenarchaeotic Group;
JdFR-11	JGI24020J35080_1000002	651	91.1	Archaea;Miscellaneous Crenarchaeotic Group;
JdFR-11	JGI24020J35080_1000015	943	87.8	Archaea;Miscellaneous Crenarchaeotic Group;
JdFR-13	JGI24019J35510_1000004	662	92.1	Archaea;Aigarchaeota;Terrestrial Hot Spring Gp(THSCG);
JdFR-14	JGI24020J35080_1000001	1,489	96.0	Archaea;Aigarchaeota;Terrestrial Hot Spring Gp(THSCG);
JdFR-17	JGI24020J35080_1002868	1,079	87.1	Archaea;Euryarchaeota;Thermoplasmata;Marine Benthic Group E;
JdFR-18	JGI24020J35080_1000023	1,474	94.1	Archaea;Euryarchaeota;Thermoplasmata;Marine Benthic Group E;
JdFR-19	JGI24020J35080_1000052	1,487	93.0	Archaea;Euryarchaeota;Methanomicrobia;Methanosarcinales;
JdFR-20	JGI24020J35080_1005442	1,473	88.0	Archaea;Euryarchaeota;Archaeoglobi;Archaeoglobales;Archaeoglobaceae;
JdFR-21	JGI24019J35510_1000058	1,271	89.6	Archaea;Euryarchaeota;Archaeoglobi;Archaeoglobales;Archaeoglobaceae;
JdFR-21	JGI24019J35510_1000173	1,353	88.9	Archaea;Euryarchaeota;Archaeoglobi;Archaeoglobales;Archaeoglobaceae;
JdFR-22	JGI24020J35080_1000953	888	91.1	Archaea;Euryarchaeota;Archaeoglobi;Archaeoglobales;Archaeoglobaceae;
JdFR-23	JGI24019J35510_1000683	893	91.1	Archaea;Euryarchaeota;Archaeoglobi;Archaeoglobales;Archaeoglobaceae;
JdFR-24	JGI24020J35080_1000512	582	92.0	Archaea;Euryarchaeota;Archaeoglobi;Archaeoglobales;Archaeoglobaceae;Archaeoglobus;
JdFR-27	JGI24020J35080_1000629	301	96.5	Archaea;Euryarchaeota;Archaeoglobi;Archaeoglobales;Archaeoglobaceae;Archaeoglobus;
JdFR-28	JGI24019J35510_1000431	301	96.5	Archaea;Euryarchaeota;Archaeoglobi;Archaeoglobales;Archaeoglobaceae;Archaeoglobus;
JdFR-31	JGI24019J35510_1000118	387	94.1	Archaea;Euryarchaeota;Archaeoglobi;Archaeoglobales;Archaeoglobaceae;
JdFR-33	JGI24019J35510_1000280	301	97.9	Archaea;Euryarchaeota;Archaeoglobi;Archaeoglobales;Archaeoglobaceae;Archaeoglobus;
JdFR-37	JGI24020J35080_1002085	707	97.0	Archaea;Euryarchaeota;Archaeoglobi;Archaeoglobales;Archaeoglobaceae;Archaeoglobus;
JdFR-39	JGI24020J35080_1000238	385	98.2	Archaea;Euryarchaeota;Archaeoglobi;Archaeoglobales;Archaeoglobaceae;Archaeoglobus;
JdFR-41	JGI24020J35080_1002674	403	94.7	Archaea;Euryarchaeota;Archaeoglobi;Archaeoglobales;Archaeoglobaceae;Archaeoglobus;
JdFR-42	JGI24020J35080_1000610	903	86.9	Archaea;Euryarchaeota;Archaeoglobi;Archaeoglobales;Archaeoglobaceae;
JdFR-43	JGI24020J35080_1001607	1,479	90.4	Archaea;Euryarchaeota;Thermoplasmata;Thermoplasmatales;20c-4;
JdFR-44	JGI24019J35510_1005882	657	94.8	Archaea;Euryarchaeota;Thermoplasmata;Thermoplasmatales;Deep Sea Hydrothermal Vent Gp 2(DHVEG-2);
JdFR-45	JGI24020J35080_1009944	1,477	92.5	Archaea;Euryarchaeota;Thermoplasmata;Thermoplasmatales;Deep Sea Hydrothermal Vent Gp 2(DHVEG-2);
JdFR-46	JGI24020J35080_1005312	1,020	93.7	Bacteria;Acetothermia;
JdFR-48	JGI24019J35510_1000483	409	94.6	Bacteria;Acetothermia;
JdFR-51	JGI24019J35510_1000234	397	96.1	Bacteria;Acetothermia;
JdFR-51	JGI24019J35510_1002932	379	98.0	Bacteria;Acetothermia;
JdFR-52	JGI24020J35080_1002589	398	96.1	Bacteria;Acetothermia;
JdFR-52	JGI24020J35080_1007827	810	96.8	Bacteria;Acetothermia;
JdFR-54	JGI24020J35080_1000047	1,512	88.1	Bacteria;Chloroflexi;Dehalococcoidia;
JdFR-56	JGI24020J35080_1000114	404	84.5	Bacteria;Chloroflexi;Dehalococcoidia;
JdFR-60	JGI24019J35510_1004897	399	91.6	Bacteria;Chloroflexi;Anaerolineae;Anaerolineales;Anaerolineaceae;uncultured;
JdFR-63	JGI24019J35510_1004359	410	87.6	Bacteria;Chloroflexi;Anaerolineae;Anaerolineales;Anaerolineaceae;uncultured;
JdFR-64	JGI24020J35080_1003996	1,515	89.0	Bacteria;Chloroflexi;
JdFR-72	JGI24020J35080_1000030	1,580	80.3	Bacteria;Thermotogae;Thermotogae;Thermotogales;Thermotogaceae;EM3;
JdFR-74	JGI24019J35510_1001968	1,465	83.9	Bacteria;Thermotogae;Thermotogae;Thermotogales;Thermotogaceae;EM3;
JdFR-76	JGI24019J35510_1001944	1,589	83.7	Bacteria;Deferribacteres;Deferribacteres Incertae Sedis;Unknown Order;Unknown Family;Caldithrix;
JdFR-78	JGI24020J35080_1000056	425	83.7	Bacteria;Aminicenantes;
JdFR-80	JGI24020J35080_1000564	413	96.5	Bacteria;Aminicenantes;
JdFR-81	JGI24020J35080_1004811	431	77.2	Bacteria;Nitrospirae;Nitrospira;Nitrospirales;Nitrospiraceae;uncultured;
JdFR-83	JGI24020J35080_1001622	377	78.3	Bacteria;Nitrospirae;Nitrospira;Nitrospirales;Nitrospiraceae;uncultured;
JdFR-84	JGI24020J35080_1004531	433	81.8	Bacteria;Nitrospirae;Nitrospira;Nitrospirales;Nitrospiraceae;uncultured;
JdFR-87	JGI24019J35510_1000862	1,592	85.9	Bacteria;Nitrospirae;Nitrospira;Nitrospirales;Nitrospiraceae;uncultured;
JdFR-88	JGI24020J35080_1000038	1,593	86.0	Bacteria;Nitrospirae;Nitrospira;Nitrospirales;Nitrospiraceae;uncultured;
JdFR-97	JGI24020J35080_1000249	1,584	94.1	Bacteria;Proteobacteria;Deltaproteobacteria;Desulfarculales;Desulfarculaceae;uncultured;
JdFR-98	JGI24019J35510_1007260	1,285	94.0	Bacteria;Proteobacteria;Deltaproteobacteria;Desulfarculales;Desulfarculaceae;uncultured;

**Table 5:** Summary of genomes from metagenomes (GFMs)

**Bin**	**Taxonomy**	**Size (Kbp)**	**Contigs**	**Genes**	**N50**	**GC (%)**	**Completeness (%)**	**Contamination (%)**	**BioSample**	**GenBank Accession**
JdFR-01	Candidatus Bathyarchaeota archaeon	200	53	262	3,811	38.2	14.5	0.9	SAMN06226318	MTLO00000000
JdFR-02	Thermococcus sp.	2,362	44	2,708	1,35,395	38.4	100.0	1.2	SAMN06226319	MTLP00000000
JdFR-03	Methanothermococcus sp.	1,476	164	1,639	11,655	33.0	91.2	1.7	SAMN06226320	MTLQ00000000
JdFR-04	Candidatus Bathyarchaeota archaeon	1,068	204	1,345	5722	41.9	77.3	1.3	SAMN06226321	MTLR00000000
JdFR-05	Candidatus Bathyarchaeota archaeon	433	101	572	4,308	42.4	41.1	0.0	SAMN06226322	MTLS00000000
JdFR-06	Candidatus Bathyarchaeota archaeon	735	72	920	24,679	38.1	37.4	2.8	SAMN06226323	MTLT00000000
JdFR-07	Candidatus Bathyarchaeota archaeon	908	43	1,061	33,770	39.1	64.5	0.7	SAMN06226324	MTLU00000000
JdFR-08	Candidatus Bathyarchaeota archaeon	919	45	1,079	28,674	38.6	52.3	2.9	SAMN06226325	MTLV00000000
JdFR-09	Candidatus Bathyarchaeota archaeon	499	16	621	39,478	39.1	28.7	0.0	SAMN06226326	MTLW00000000
JdFR-10	Candidatus Bathyarchaeota archaeon	1,818	57	2,051	94,071	51.4	97.1	6.5	SAMN06226327	MTLX00000000
JdFR-11	Candidatus Bathyarchaeota archaeon	1,726	12	1,932	2,51,681	52.0	100.0	0.9	SAMN06226328	MTLY00000000
JdFR-12	Crenarchaeota archaeon	301	65	372	3,767	36.1	11.0	0.0	SAMN06226329	MTLZ00000000
JdFR-13	Candidatus Geothermarchaeota archaeon	1,672	4	1,780	4,88,929	37.6	93.2	1.0	SAMN06226330	MTMA00000000
JdFR-14	Candidatus Geothermarchaeota archaeon	1,635	6	1,722	4,62,362	41.9	94.2	17.5	SAMN06226331	MTMB00000000
JdFR-15	Methanopyri archaeon	208	54	273	3,688	36.0	14.5	0.0	SAMN06226332	MTMC00000000
JdFR-16	Candidatus Hydrothermarchaeota archaeon	1,353	241	1,714	6,267	50.4	31.9	13.6	SAMN06226333	MTMD00000000
JdFR-17	Candidatus Hydrothermarchaeota archaeon	2,178	344	2,766	7,687	50.1	53.9	25.2	SAMN06226334	MTME00000000
JdFR-18	Candidatus Hydrothermarchaeota archaeon	2,062	22	2,328	1,49,032	39.1	98.1	1.9	SAMN06226335	MTMF00000000
JdFR-19	Methanomicrobia archaeon	1,289	27	1,594	78,121	43.2	96.7	0.0	SAMN06226336	MTMG00000000
JdFR-20	Euryarchaeota archaeon	1,610	209	2,109	8,881	41.1	89.4	7.7	SAMN06226337	MTMH00000000
JdFR-21	Euryarchaeota archaeon	1,417	22	1,724	1,02,423	41.2	96.1	0.7	SAMN06226338	MTMI00000000
JdFR-22	Archaeoglobus sp.	2,063	130	2,360	20,363	39.7	98.7	0.0	SAMN06226339	MTMJ00000000
JdFR-23	Archaeoglobus sp.	1,629	101	1,924	25,841	40.0	64.1	5.2	SAMN06226340	MTMK00000000
JdFR-24	Archaeoglobus sp.	2,702	154	3,011	48,758	38.2	100.0	8.2	SAMN06226341	MTML00000000
JdFR-25	Archaeoglobus sp.	885	31	1,007	65,531	39.9	28.0	0.0	SAMN06226342	MTMM00000000
JdFR-26	Archaeoglobus sp.	645	21	713	1,41,267	40.2	23.4	2.8	SAMN06226343	MTMN00000000
JdFR-27	Archaeoglobus sp.	2,360	67	2,680	82,469	40.6	94.8	3.3	SAMN06226344	MTMO00000000
JdFR-28	Archaeoglobus sp.	752	14	829	97,698	40.4	26.8	0.0	SAMN06226345	MTMP00000000
JdFR-29	Archaeoglobus sp.	738	185	967	3,748	44.9	24.3	2.3	SAMN06226346	MTMQ00000000
JdFR-30	Archaeoglobus sp.	1,100	55	1,276	33,048	39.5	44.4	3.9	SAMN06226347	MTMR00000000
JdFR-31	Archaeoglobus sp.	2,351	103	2,752	43,068	42.1	92.8	6.6	SAMN06226348	MTMS00000000
JdFR-32	Archaeoglobus sp.	1,967	120	2,225	24,104	41.8	99.4	0.0	SAMN06226349	MTMT00000000
JdFR-33	Archaeoglobus sp.	479	89	593	5,918	40.3	10.3	0.0	SAMN06226350	MTMU00000000
JdFR-34	Archaeoglobus sp.	1,088	88	1,246	18,271	41.3	58.8	0.0	SAMN06226351	MTMV00000000
JdFR-35	Archaeoglobus sp.	1,703	167	2,029	14,968	41.2	91.5	0.7	SAMN06226352	MTMW00000000
JdFR-36	Archaeoglobus sp.	581	75	732	9,486	40.6	23.5	0.7	SAMN06226353	MTMX00000000
JdFR-37	Archaeoglobus sp.	1,972	52	2,248	64,398	44.7	94.8	0.7	SAMN06226354	MTMY00000000
JdFR-38	Archaeoglobus sp.	1,025	99	1,208	13,482	44.5	43.2	0.0	SAMN06226355	MTMZ00000000
JdFR-39	Archaeoglobus sp.	2,279	93	2,743	55,279	43.8	100.0	0.7	SAMN06226356	MTNA00000000
JdFR-40	Archaeoglobus sp.	530	76	671	8,743	42.8	17.8	0.0	SAMN06226357	MTNB00000000
JdFR-41	Archaeoglobus sp.	1,752	103	1,921	25992	42.3	95.9	0.0	SAMN06226358	MTNC00000000
JdFR-42	Archaeoglobi archaeon	2,149	42	2,479	70,809	40.3	99.8	2.0	SAMN06226359	MTND00000000
JdFR-43	Thermoplasmatales archaeon	1,231	219	1,425	6,292	37.5	78.5	0.0	SAMN06226360	MTNE00000000
JdFR-44	Candidatus Aciduliprofundum sp.	519	139	527	3,618	55.3	30.0	0.0	SAMN06226361	MTNF00000000
JdFR-45	Candidatus Aciduliprofundum sp.	1,250	161	1,454	9,500	57.9	92.4	1.6	SAMN06226362	MTNG00000000
JdFR-46	Candidatus Acetothermia bacterium	1,088	254	1,259	4,279	65.9	54.0	6.3	SAMN06226363	MTNH00000000
JdFR-47	Candidatus Acetothermia bacterium	1,622	230	1,811	8,291	63.7	82.3	1.7	SAMN06226364	MTNI00000000
JdFR-48	Candidatus Acetothermia bacterium	1,893	62	1,992	56,879	61.1	91.5	0.0	SAMN06226365	MTNJ00000000
JdFR-49	Candidatus Acetothermia bacterium	1,699	312	2,003	5,976	59.9	68.4	2.5	SAMN06226366	MTNK00000000
JdFR-50	Candidatus Acetothermia bacterium	1,292	255	1,516	5,492	62.2	58.6	0.0	SAMN06226367	MTNL00000000
JdFR-51	Candidatus Acetothermia bacterium	1,764	185	1,919	13,057	63.0	88.1	3.4	SAMN06226368	MTNM00000000
JdFR-52	Candidatus Acetothermia bacterium	1,711	155	1,870	17,111	62.7	86.4	4.2	SAMN06226369	MTNN00000000
JdFR-53	Unknown bacterium	346	76	434	4,902	38.4	33.6	2.0	SAMN06226370	MTNO00000000
JdFR-54	Dehalococcoides sp.	1,691	67	1,743	54,623	59.9	77.9	2.3	SAMN06226371	MTNP00000000
JdFR-55	Dehalococcoides sp.	546	132	599	4,081	62.8	16.9	4.3	SAMN06226372	MTNQ00000000
JdFR-56	Dehalococcoides sp.	1,798	95	1,964	28,676	57.1	87.7	1.0	SAMN06226373	MTNR00000000
JdFR-57	Dehalococcoides sp.	776	207	951	3,716	57.6	32.8	2.0	SAMN06226374	MTNS00000000
JdFR-58	Dehalococcoides sp.	908	203	1,152	4,219	57.4	46.0	3.0	SAMN06226375	MTNT00000000
JdFR-59	Chloroflexi bacterium	2,039	527	2,194	3,822	61.0	36.6	1.8	SAMN06226376	MTNU00000000
JdFR-60	Anaerolineales bacterium	863	239	992	3,541	64.5	27.7	0.2	SAMN06226377	MTNV00000000
JdFR-61	Anaerolineales bacterium	2,907	380	2,909	8,915	64.2	81.5	16.9	SAMN06226378	MTNW00000000
JdFR-62	Anaerolineales bacterium	906	183	1,068	5,571	52.3	31.7	2.0	SAMN06226379	MTNX00000000
JdFR-63	Anaerolineales bacterium	1,318	282	1,521	5,066	52.4	45.7	1.8	SAMN06226380	MTNY00000000
JdFR-64	Anaerolineales bacterium	2,358	468	2,680	5,560	52.6	73.7	5.9	SAMN06226381	MTNZ00000000
JdFR-65	Unknown bacterium	937	193	1,101	5,093	42.2	53.5	3.6	SAMN06226382	MTOA00000000
JdFR-66	Unknown bacterium	1,839	224	2,056	11,163	40.4	83.6	7.8	SAMN06226383	MTOB00000000
JdFR-67	Unknown bacterium	874	172	978	5,273	41.0	41.8	1.8	SAMN06226384	MTOC00000000
JdFR-68	Thermoanaerobacterales bacterium	2,976	458	3,396	7,798	39.7	90.7	2.9	SAMN06226385	MTOD00000000
JdFR-69	Peptococcaceae bacterium	1,780	36	1,865	1,11,790	61.1	97.6	0.6	SAMN06226386	MTOE00000000
JdFR-70	Unknown bacterium	921	214	596	4,521	35.8	57.2	1.1	SAMN06226387	MTOF00000000
JdFR-71	Candidatus Hydrothermae bacterium	1,702	18	1,729	1,42,680	34.8	81.4	0.0	SAMN06226388	MTOG00000000
JdFR-72	Candidatus Hydrothermae bacterium	2,060	19	2,026	1,76,033	34.7	91.5	0.0	SAMN06226389	MTOH00000000
JdFR-73	Candidatus Hydrothermae bacterium	1,305	257	1,371	5,573	32.8	37.0	5.2	SAMN06226390	MTOI00000000
JdFR-74	Candidatus Hydrothermae bacterium	1,801	267	1,861	8,536	32.9	47.9	0.6	SAMN06226391	MTOJ00000000
JdFR-75	Candidatus Hydrothermae bacterium	789	171	979	4,417	32.1	40.0	0.0	SAMN06226392	MTOK00000000
JdFR-76	Deferribacteres bacterium	3,100	557	3,087	5,995	52.3	77.9	0.3	SAMN06226393	MTOL00000000
JdFR-77	Candidatus Aminicenantes bacterium	2,326	200	2,380	16,386	30.5	82.6	8.7	SAMN06226394	MTOM00000000
JdFR-78	Candidatus Aminicenantes bacterium	2,530	42	2,463	1,14,879	32.5	93.9	2.6	SAMN06226395	MTON00000000
JdFR-79	Candidatus Aminicenantes bacterium	2,046	32	1,995	94,220	32.7	74.4	1.7	SAMN06226396	MTOO00000000
JdFR-80	Candidatus Aminicenantes bacterium	2,915	113	2,726	57,497	44.5	92.9	5.1	SAMN06226397	MTOP00000000
JdFR-81	Nitrospirae bacterium	2,050	95	2,117	39,421	48.0	94.6	1.8	SAMN06226398	MTOQ00000000
JdFR-82	Nitrospirae bacterium	736	154	875	5,174	43.8	47.0	4.4	SAMN06226399	MTOR00000000
JdFR-83	Nitrospirae bacterium	1,165	177	1,310	8,103	42.1	63.9	9.1	SAMN06226400	MTOS00000000
JdFR-84	Nitrospirae bacterium	1,526	342	1,833	4,439	39.9	34.4	8.4	SAMN06226401	MTOT00000000
JdFR-85	Nitrospirae bacterium	2,326	47	2,399	78,042	41.4	98.2	1.8	SAMN06226402	MTOU00000000
JdFR-86	Nitrospirae bacterium	2,103	25	2,166	1,24,076	45.2	98.2	0.8	SAMN06226403	MTOV00000000
JdFR-87	Nitrospirae bacterium	1,861	58	2,005	51,364	62.5	98.2	1.8	SAMN06226404	MTOW00000000
JdFR-88	Nitrospirae bacterium	1,858	22	1,983	1,31,863	62.8	98.2	0.9	SAMN06226405	MTOX00000000
JdFR-89	Caulobacteraceae bacterium	4,055	650	4,284	7,375	67.8	81.0	4.1	SAMN06226406	MTOY00000000
JdFR-90	Cupriavidus sp.	2,666	703	3,105	3,723	62.8	40.1	2.0	SAMN06226407	MTOZ00000000
JdFR-91	Pseudomonas sp.	3,770	358	3,583	15,126	60.9	61.0	0.3	SAMN06226408	MTPA00000000
JdFR-92	Pseudomonas sp.	1,611	457	1,865	3,440	59.3	27.0	0.4	SAMN06226409	MTPB00000000
JdFR-93	Pseudomonas sp.	2,230	225	2,227	13,223	59.8	35.3	0.5	SAMN06226410	MTPC00000000
JdFR-94	Acinetobacter sp.	369	117	478	3,101	37.9	11.2	0.0	SAMN06226411	MTPD00000000
JdFR-95	Desulfarculaceae bacterium	2,808	499	2,872	6,282	68.3	68.5	0.9	SAMN06226412	MTPE00000000
JdFR-96	Desulfarculaceae bacterium	1,393	312	1,551	4,552	58.3	42.1	2.6	SAMN06226413	MTPF00000000
JdFR-97	Desulfarculaceae bacterium	4,103	127	3,814	54,652	57.0	96.8	2.6	SAMN06226414	MTPG00000000
JdFR-98	Desulfarculaceae bacterium	933	224	1,057	3,945	57.7	20.9	2.6	SAMN06226415	MTPG00000000
