# Corrigendum: Metagenome sequencing and 98 microbial genomes from Juan de Fuca Ridge flank subsurface fluids

**DOI:** 10.1038/sdata.2017.80

**Published:** 2017-07-04

**Authors:** Sean P. Jungbluth, Jan P. Amend, Michael S. Rappé

**Keywords:** Metagenomics, Marine biology, Genome informatics, Microbial ecology, DNA sequencing

*Scientific Data* 4:170037 doi: 10.1038/sdata.2017.37 (2017) ; Published 28 March 2017; Updated 4 July 2017

Table 5 and Supplementary Table 1 contain an error in the Genbank accession provided for bin JdFR-98. The correct accession number is “MTPH00000000”, not “MTPG00000000”.

