# Supplementary material for: Metagenome sequencing and 98 microbial genomes from Juan de Fuca Ridge flank subsurface fluids
Source: Sci Data. 2017 Mar 28;4:170037. doi: 10.1038/sdata.2017.37 (PMC5369317; doi:10.1038/sdata.2017.37)
Supplement: Supplementary Figures [file sdata201737-s2.pdf]

**Table of Contents**


Figure S1: Archaeal phylogenomic relationships Page 1

Figure S2: Bacterial phylogenomic relationships Page 2

Figure S3: Genome binning with ESOM Page 3

Figure S4: Genome bin completeness and contamination Page 4

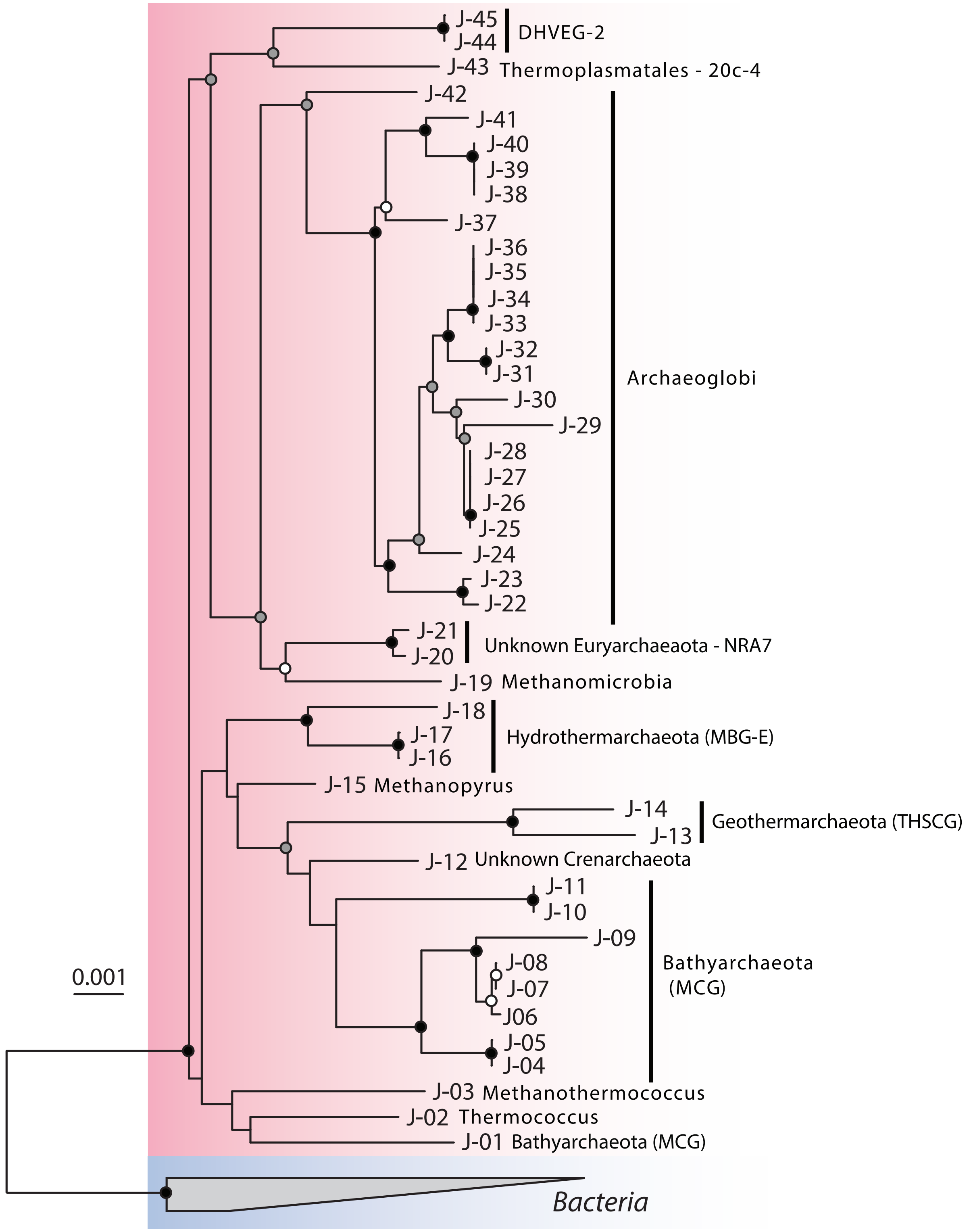


Figure S1. Phylogenomic relationships between archaeal GFMs > 10% complete identified in metagenomes from deep subseafloor crustal fluids of boreholes U1362A and U1362B. Archaeal GFMs found in this study were used as the outgroup. The scale bar corresponds to 0.001 substitutions per amino acid position. Black (100%), gray (≥ 80%), and white (≥ 50%) circles indicate nodes with bootstrap support, from 100 replicates. All bins are abbreviated "J" for "JdFR".

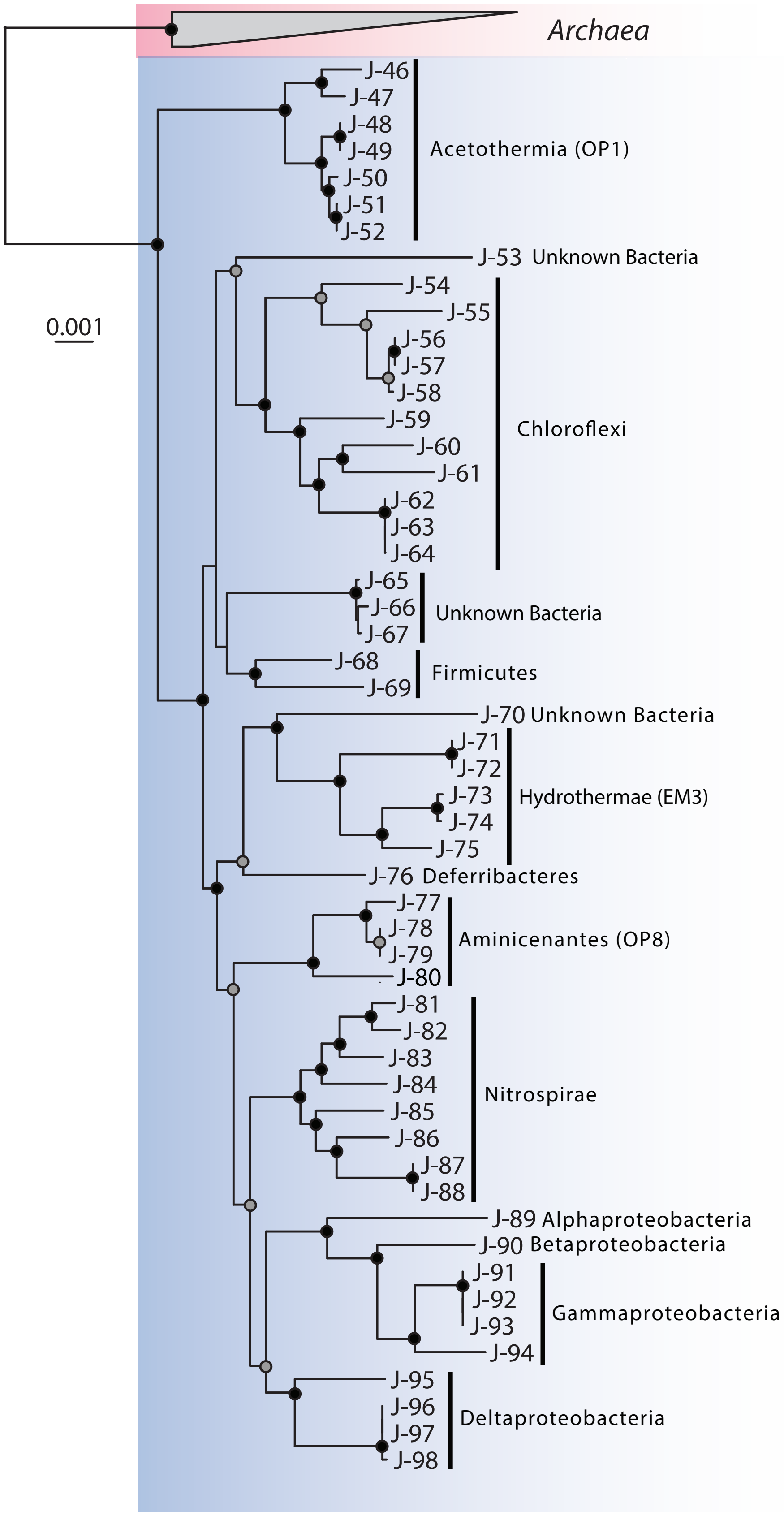


Figure S2. Phylogenomic relationships between bacterial GFMs > 10% complete identified in metagenomes from deep subseafloor crustal fluids of boreholes U1362A and U1362B. Bacterial GFMs found in this study were used as the outgroup. Other information as in Figure S1.

a)

b)

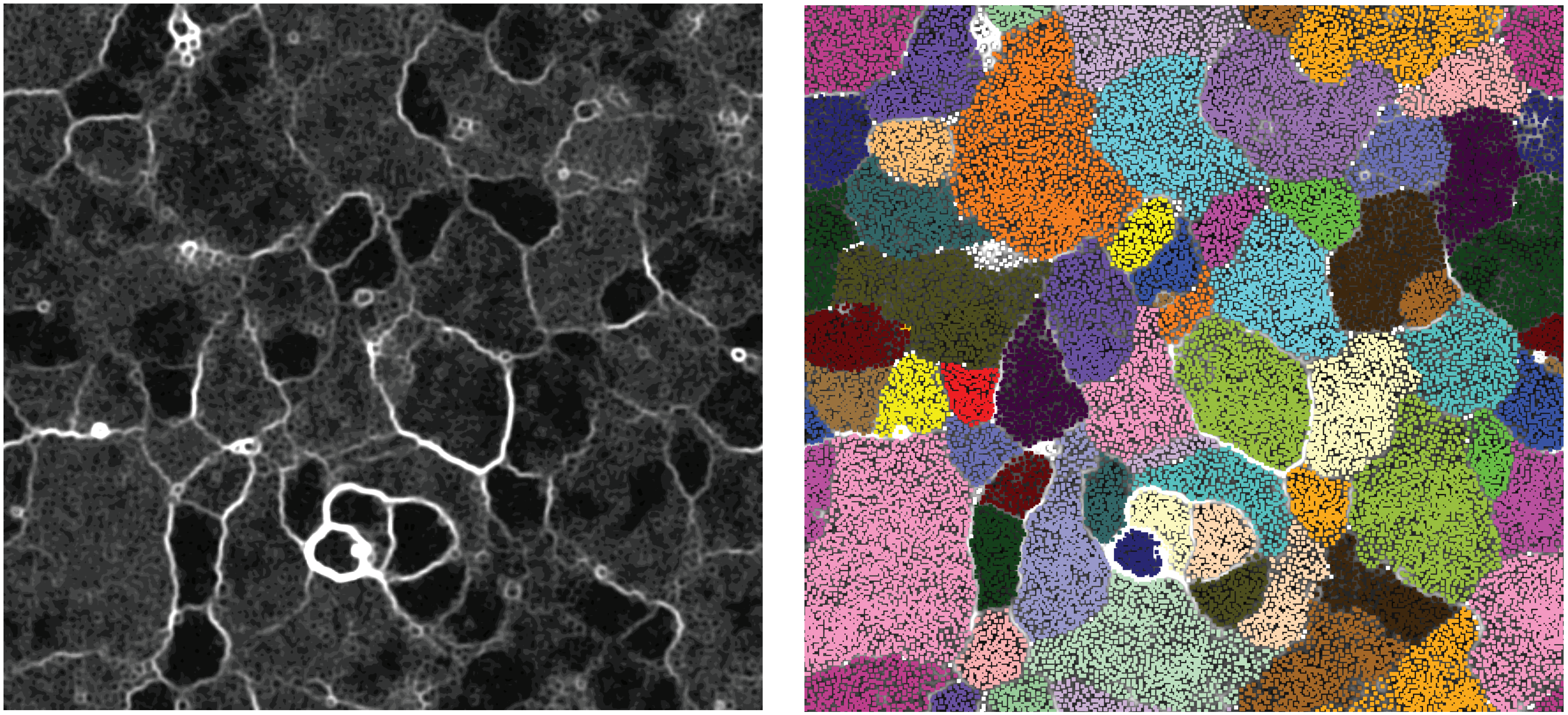

Figure S3. Assignment of contigs from CORK borehole fluid metagenomes using ESOM implemented with tetranucleotide frequencies and differential coverage. The ESOM is shown (A) before and (B) after identification of GFMs. Each point represents a contig and identified bins have a non-white color.

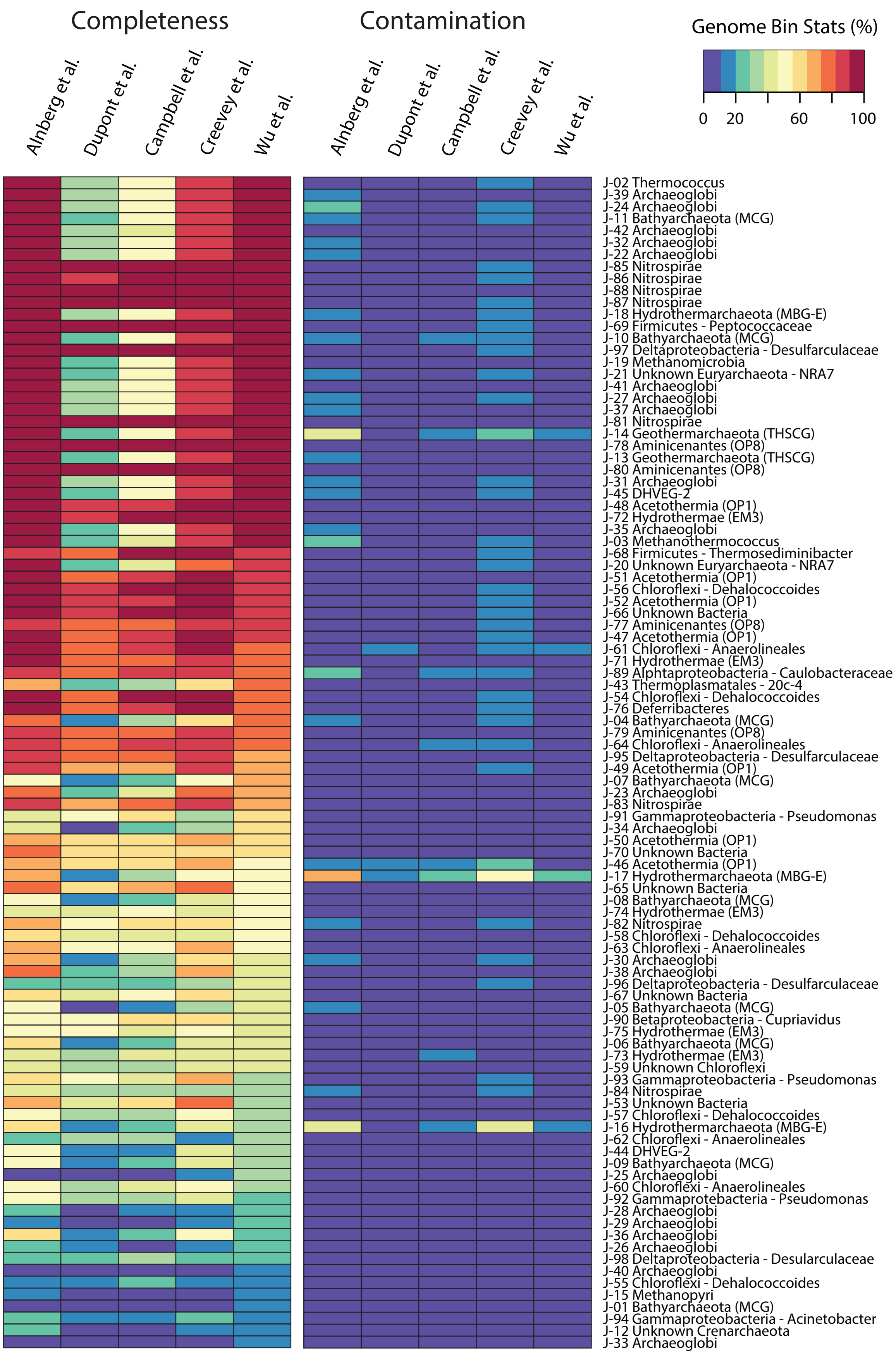


Fig S4. Overview of GFM completeness and contamination and calculated average using five different marker gene sets. All bins are abbreviated "J" for "JdFR."
